# Fentanyl Disrupts Vagal Control of Airway Tone to Induce Transient Obstruction

**DOI:** 10.1111/apha.70119

**Published:** 2025-10-15

**Authors:** Riley R. Parks, Marissa J. Andersen, Mackenna L. Hatfield, Nicholas J. Burgraff

**Affiliations:** ^1^ Department of Comparative Biosciences University of Wisconsin‐Madison Madison Wisconsin USA

## Abstract

**Aim:**

Opioid‐induced respiratory depression (OIRD) is the primary cause of death in opioid overdose, resulting from both suppressed respiratory rhythm and increased airway and thoracic rigidity that compromise ventilation and resuscitation. While the effect(s) of opioids on central rhythm‐generating circuits are well documented, the mechanisms leading to airway obstruction remain poorly understood. Here, we investigated the hypothesis that enhanced vagal parasympathetic output contributes to fentanyl‐induced airway disruption.

**Methods:**

In urethane‐anesthetized mice, diaphragm electromyography (EMG), respiratory airflow, and vagus nerve activity were recorded in‐vivo before and after intraperitoneal fentanyl administration (500 μg/kg). The effects of bilateral vagotomy, atropine administration, and intracisternal naloxone were evaluated to determine the contribution of vagal pathways and central opioid receptor mechanisms.

**Results:**

Fentanyl caused a characteristic slowing of respiratory rate accompanied by a compensatory increase in tidal volume, but also produced a transient delay between diaphragm activation and airflow onset, consistent with airway obstruction. This delay was abolished by bilateral vagotomy or atropine and reversed by intracisternal naloxone, implicating central vagal mechanisms. Vagal electroneurograms showed increased tonic multiunit activity and enhanced large‐amplitude single‐unit firing, particularly within efferent fibers, together with a loss of normal inspiratory phase‐locking. The magnitude of tonic vagal activation strongly correlated with the severity of airway disruption.

**Conclusions:**

Fentanyl disrupts respiratory‐autonomic integration by enhancing parasympathetic vagal drive, producing a central, opioid receptor‐mediated mechanism of airway constriction. Targeting vagal pathways may therefore represent a promising adjunctive strategy for improving airway patency and ventilatory recovery during opioid overdose reversal.

**Summary**

- Fentanyl induces transient airway obstruction in part through increased central vagal output, disrupting the normal timing between diaphragm activation and inspiratory airflow during overdose.
- This obstruction is abolished by vagotomy or atropine and reversed by intracisternal naloxone, indicating a centrally mediated, opioid receptor‐dependent mechanism driving bronchoconstriction.
- Vagus nerve activity becomes desynchronized from the respiratory cycle, with increased tonic firing and loss of inspiratory phase‐locking, highlighting a breakdown in respiratory‐autonomic coordination.

## 1 Introduction

The production and widespread availability of prescription and illicit opioids has created an epidemic resulting in the deaths of millions worldwide [1]. Opioid overdose‐related deaths are primarily the result of opioid‐induced respiratory depression (OIRD) [2, 3, 4, 5, 6, 7, 8, 9] a phenomenon in which breathing becomes slow and shallow, leading to insufficient ventilation to sustain life [10]. Unlike traditional opioids, fentanyl not only depresses respiratory neural circuits but also induces profound muscular rigidity, including stiffening of the airways and thoracic muscles, a phenomenon termed Wooden Chest Syndrome (WCS) [11, 12, 13, 14, 15, 16]. This rigidity impairs both airflow through the upper and lower airways and impairs mechanical ventilation by the diaphragm, compounding the risk of death during overdose [13]. Additionally, these effects compound the severity of acute resuscitation by first responders, rendering patients unable to be bag‐ventilated and difficult to intubate. While fentanyl's effects on respiratory rhythm have been well studied, the effects on the airways represent a critical but underexplored contributor to opioid‐induced respiratory depression.

Airway patency is dynamically regulated by a complex interplay of sensory (afferent) and motor (efferent) neural circuits, with the vagus nerve serving as the primary pathway for parasympathetic control of the respiratory tract [17, 18, 19, 20, 21]. Vagal afferent fibers originate from the nodose and jugular ganglia and innervate the airways extensively, terminating in airway smooth muscle, epithelium, vasculature, and ganglia [22, 23, 24, 25]. These afferents include rapidly adapting receptors (RARs) and slowly adapting receptors (SARs), myelinated A fibers that are sensitive to lung inflation dynamics, as well as unmyelinated C fibers, which act as chemosensors responsive to irritants, inflammatory mediators, and changes in the tissue environment [19, 26, 27]. Activation of airway afferents can trigger reflexes that modulate breathing patterns, bronchoconstriction, mucus secretion, and cough, largely through their integration within the nucleus tractus solitarius (NTS) in the brainstem [25, 28, 29, 30, 31].

On the efferent side, preganglionic parasympathetic neurons originate predominantly in the nucleus ambiguus and to a lesser extent the dorsal motor nucleus of the vagus (DMV) [18, 21, 32, 33]. These fibers project to parasympathetic ganglia embedded within the airway walls, where they synapse onto postganglionic neurons that innervate airway smooth muscle (ASM), vasculature, and secretory glands. Acetylcholine released from these postganglionic fibers activates M3 muscarinic receptors on ASM to promote contraction and bronchoconstriction, establishing a basal bronchomotor tone [17, 34]. Although M2 receptors are more abundant on ASM, their primary role is thought to be inhibitory at presynaptic sites, regulating acetylcholine release rather than directly driving contraction [35, 36].

This dual afferent‐efferent organization makes the vagus nerve a critical regulator of airway resistance. Reflex loops initiated by afferent activation, including responses to airway stretch, mechanical stimulation, or chemical irritation, feedback to adjust efferent output to airway smooth muscle [22, 37, 38]. Disruption of this finely tuned balance can markedly alter airway patency.

Importantly, opioid receptors, particularly μ‐opioid receptors (MORs), are highly expressed within key regions controlling vagal output, including the nucleus ambiguus, DMV, and NTS [39]. Additionally, MORs have been identified on vagal afferent and efferent fibers themselves [40, 41]. This anatomical and molecular framework suggests that opioids, especially potent agents like fentanyl, could modulate airway tone through multiple mechanisms: suppressing or enhancing vagal afferent signaling, altering central parasympathetic drive, or directly affecting peripheral parasympathetic output at the level of airway ganglia or smooth muscle.

Prior studies support these possibilities. For example, Cohendy et al. [42] demonstrated in humans that fentanyl significantly increased total inspiratory resistance (Rmax) by elevating both airway resistance (Raw) and non‐Newtonian resistance (ΔR), with the Raw component reversed by atropine, implicating vagally mediated bronchoconstriction. At the central level, Hajiha et al. [43] showed that local fentanyl delivery into the hypoglossal motor nucleus suppressed genioglossus muscle activity via MORs, independent of muscarinic receptor involvement, highlighting direct opioid modulation of motor nuclei controlling upper airway patency. Together, these findings demonstrate that fentanyl can act at both peripheral and central sites to disrupt airway function through vagal and motor control pathways, consistent with our proposed mechanism.

Given fentanyl's capacity to cause muscular rigidity and its known modulation of neural excitability through both receptor‐mediated and noncanonical pathways, vagal dysfunction presents a plausible mechanism for the airway constriction observed during overdose. Understanding how fentanyl impacts this integrated vagal control of airway function is essential for elucidating new therapeutic targets to mitigate opioid‐induced respiratory depression and airway obstruction.

## 2 Methods

### 2.1 Ethics Statement

Experiments were performed on adult male and female C57BL/6J mice bred at the University of Wisconsin‐Madison in accordance with the University of Wisconsin‐Madison's Institute Animal Care and Use Committee (Protocol V006882) and the National Institutes of Health guidelines. Adult mice weighed between 17.2 and 29 g and were group‐housed and given access to food and water ad libitum. Light and dark cycles were maintained at 12 h each.

### 2.2 In Vivo Electromyographic and Neural Recordings

Adult mice were initially anesthetized using isoflurane (3%), followed by intraperitoneal administration of urethane (1.5 mg/kg). During the experimental procedures, mice were placed in a supine position on a heated surgical table to maintain body temp at ~37°C and spontaneously breathed 100% O2 throughout the experiment. Airflow was assessed using a custom pneumotachograph [44]. Briefly, a custom mask was placed over the snout. Rigid tubing was affixed to the mask with two holes drilled ~1 cm apart in series. Between the holes, there was a wire mesh, and each hole connected to a differential pressure transducer (Buxco). Flow was calibrated following the study by injecting a constant flow from a calibrated flowmeter (Cole Palmer). Breathing rate is defined as the number of breaths taken per minute, and tidal volume as the integral of inspiratory flow per breath. Diaphragm muscle activity was monitored using electromyography (EMG) electrodes (A‐M Systems) inserted into the lateral diaphragm. EMG signals were sampled at a rate of 40 kHz, amplified by 10 k, filtered (500 Hz high‐pass, 300 Hz low‐pass), and analyzed in LabChart.

For vagus nerve recordings, the cervical vagus nerve was dissected and separated from the carotid artery. A bipolar, silver chloride hook electrode was then placed under the nerve. All surrounding tissue was removed from the vagus nerve to ensure minimization of EMG bleedthrough. After proper placement, mineral oil was added to prevent drying of the vagus during the experiment. Neural signals were sampled at a rate of 40 kHz, amplified by 10 k, filtered (5 kHz high‐pass, 300 Hz low‐pass), and recorded in LabChart. To confirm neural spike activity and isolate EMG bleedthrough [45, 46] 2% Lidocaine was administered via injection to the exposed nerve at the end of the study.

Fentanyl was administered at 500 μg/kg from a stock solution of 50 μg/mL (Hikma). The dose for fentanyl was selected based on previous reports [44] to produce comparable symptoms to an acute overdose and maintain consistent respiratory depression throughout the duration of the experiment. All experimental trials were terminated by an overdose of isoflurane, followed by cervical dislocation.

After completion of the intact vagus nerve recordings, the same procedure as above was repeated to obtain the efferent and afferent vagus nerve activity. However, after placing the exposed nerve on the hook electrodes, microscissors were used to cut the vagus nerve proximal or distal to the hook electrode. For efferent recordings, the cut was performed distal to the most inferior hook of the electrode. For afferent recordings, the cut was performed proximal to the most superior hook of the electrode. This allowed for the nerve portion of interest to remain placed across both hooks throughout the duration of the recording. Mineral oil was once again placed onto the exposed nerve half to prevent drying, and lidocaine was applied at the end of the study to confirm neural spike activity.

### 2.3 Intracisternal Injections

To assess the role of central opioid receptor signaling in fentanyl‐induced airway obstruction, naloxone was administered via intracisternal injection following the onset of obstruction. Naloxone hydrochloride (10 mg/kg) was prepared from a 25 mg/mL stock solution (Sigma‐Aldrich) and delivered using a 10 μL Hamilton microsyringe. Mice were briefly positioned with the head flexed to expose the cisterna magna, and the needle was carefully advanced into the cisternal space under visual guidance. A total volume of 5–10 μL was slowly injected over 1 min. Proper placement was confirmed by minimal resistance during injection and the absence of subcutaneous swelling. Airway parameters were continuously recorded before and after naloxone administration to assess the reversal of fentanyl‐induced obstruction.

### 2.4 Data Analysis

Analysis of respiratory parameters was completed using LabChart (ADInstruments). Spike sorting of vagus nerve activity was performed using Offline Sorter V4 (Plexon). Raw nerve signals were band‐pass filtered using a Butterworth filter (300–3000 Hz, 4th order). Spike detection thresholds were set above the level of residual activity following lidocaine application to ensure exclusion of noise and non‐neuronal events. Detected spikes were clustered using template‐based sorting, allowing for separation of putative single units based on waveform morphology and principal component space. Sorted units were then exported and subsequently analyzed in NeuroExplorer (Plexon) for further spike timing, perievent activity‐based plots, and breath phase‐aligned analyses.

### 2.5 Statistical Analysis

All statistical analyses were conducted using GraphPad Prism or custom scripts written in Python. Ventilatory parameters (respiratory rate, tidal volume, and minute ventilation) were analyzed using one‐way repeated measures ANOVA with Dunnett's multiple comparisons test to assess changes across time relative to baseline. Data were binned into 5‐min intervals for statistical comparison. Small‐amplitude vagal multiunit activity (MUA) was compared before and after fentanyl administration using paired two‐tailed Student's *t*‐tests. Large‐amplitude single‐unit spike activity was assessed using two‐way repeated measures ANOVA with Holm–Šidák multiple comparisons, with factors for respiratory phase (binned across the normalized breath cycle) and drug condition (baseline, fentanyl, and lidocaine). Spearman's rank correlation coefficients (ρ) were used to assess the relationship between vagal activity (single‐unit spike frequency or MUA amplitude) and the severity of fentanyl‐induced airflow obstruction, measured as the latency between diaphragm EMG onset and inspiratory airflow. Statistical significance of Spearman's ρ values was determined by calculating two‐tailed *p*‐values for each correlation. In volcano plots, significance thresholds were set at *p* < 0.05, and statistical significance was visualized.

## 3 Results

### 3.1 Fentanyl Causes Opioid‐Induced Respiratory Depression (OIRD)

**FIGURE 1:**
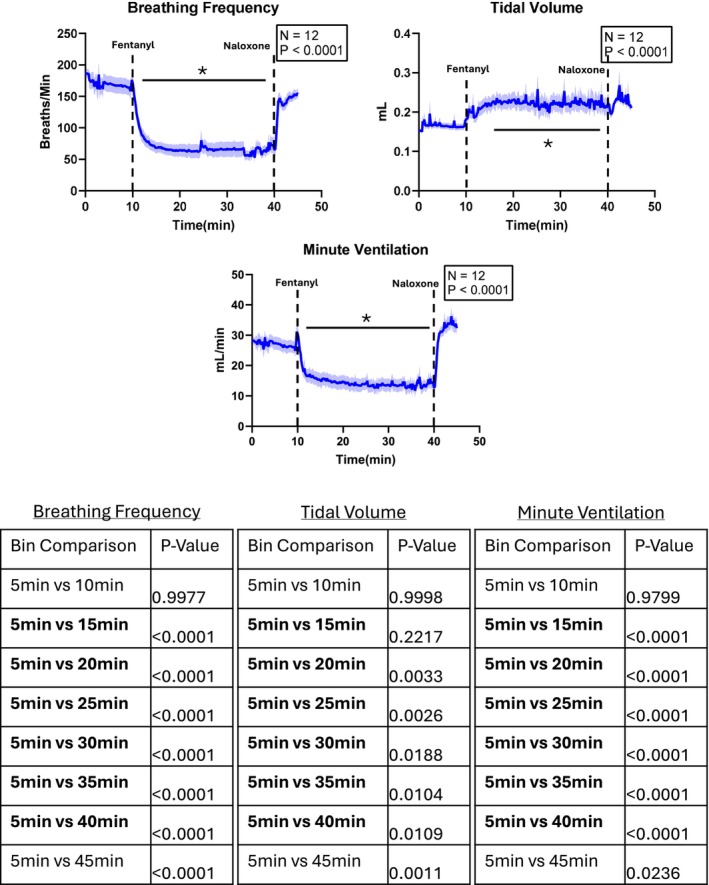
Analysis of respiration in mice following fentanyl injection. Intraperitoneal (IP) injection of fentanyl (500 μg/kg) resulted in a reduction of breathing rate by around 50% and an increase in tidal volume that ultimately resulted in decreased overall ventilation. These changes in respiratory parameters are characteristic of opioid‐induced respiratory depression (OIRD) and held steady over the course of the 30 min trial. OIRD could be reversed through IP naloxone administration (10 mg/kg). 5‐min binned data are shown in the tables below for statistical analysis across time (*N* = 12) with a one‐way RM ANOVA. Bin times represent the end of the 5 min bin (ex. “5 min” = 0‐5 min). Bolded values represent fentanyl exposure. Baseline variables, prior to fentanyl administration are shown as “5 min” and “10 min”. * represents *p* < 0.05.

Fentanyl is well‐known to suppress ventilatory activity [5, 44, 47, 48, 49, 50]. To confirm this in our model, we assessed respiratory parameters following intraperitoneal (IP) injection of fentanyl at 500 μg/kg (Figure 1). Consistent with prior reports, fentanyl administration led to a nearly 50% reduction in respiratory rate (*N* = 12). In addition, we observed a significant and sustained increase in tidal volume that persisted throughout the 30‐min post‐injection period. Together, these changes resulted in an approximate 50% decrease in minute ventilation over the duration of the 30‐min fentanyl period. Data in Figure 1 are plotted in 10‐s bins to illustrate the temporal pattern of respiratory suppression. For statistical analysis, ventilatory parameters were also averaged into 5‐min bins, as shown in the accompanying table, to facilitate comparison across timepoints.

### 3.2 Fentanyl Enhances Excitability in Tonic Vagus Nerve Activity

**FIGURE 2:**
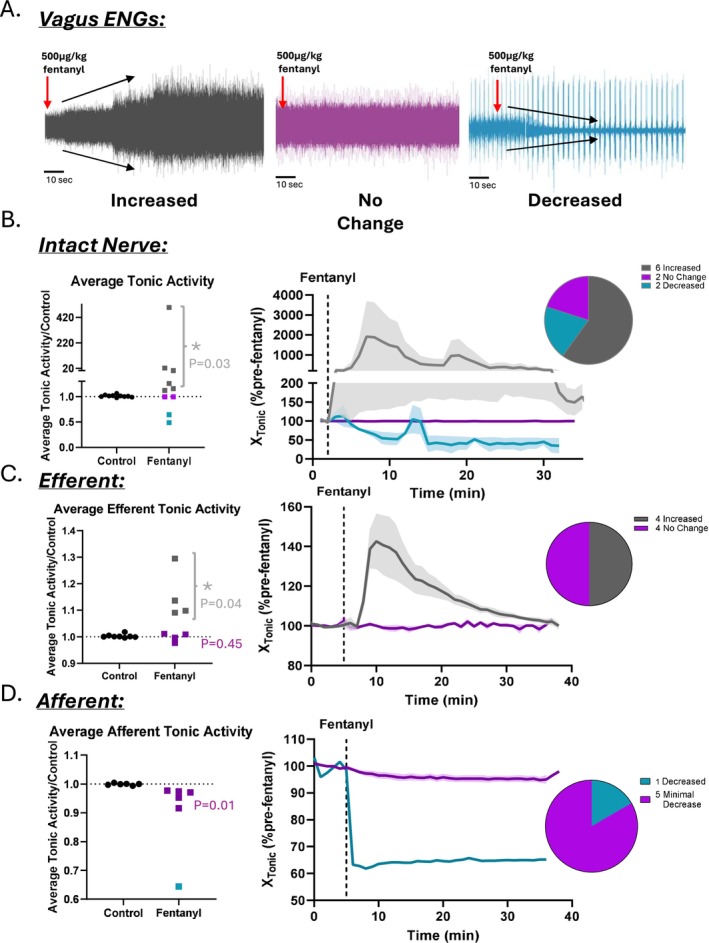
Fentanyl differentially excites and inhibits tonic vagus nerve fibers. (A) Representative tonic vagal ENG recordings illustrate three distinct patterns of response following intraperitoneal fentanyl administration (500 μg/kg). (B) In intact vagus nerve recordings, 60% of animals exhibited an increase in tonic activity (*N* = 6/10, *p* = 0.03), while 20% showed no change (*N* = 2/10) and 20% showed a decrease (*N* = 2/10) compared to baseline activity. On the left, average tonic activity is plotted across the 10‐min pre‐fentanyl control period and the 30‐min post‐fentanyl period. On the right, the mean trace of each response subtype (increase, no change, decrease) is shown with the corresponding SEM shaded. (C) In recordings from the proximal (efferent) stump following vagotomy, 50% of animals showed an increase in tonic activity (*N* = 4/8, *p* = 0.04), while the remaining 50% exhibited no change (*N* = 4/8, *p* = 0.45). (D) In recordings from the distal (afferent) stump, 83% of animals showed a statistically significant (*N* = 5/6, *p* = 0.01), but non‐appreciable change in tonic activity, while 17% (*N* = 1/6) exhibited a decrease compared to pre‐fentanyl baseline. As in panel B, average tonic activity over time is shown on the left, and mean traces for each response group are shown on the right with SEM shading. * represents *p* < 0.05. Data were assessed using paired two‐tailed Student's *t*‐tests.

**FIGURE 3:**
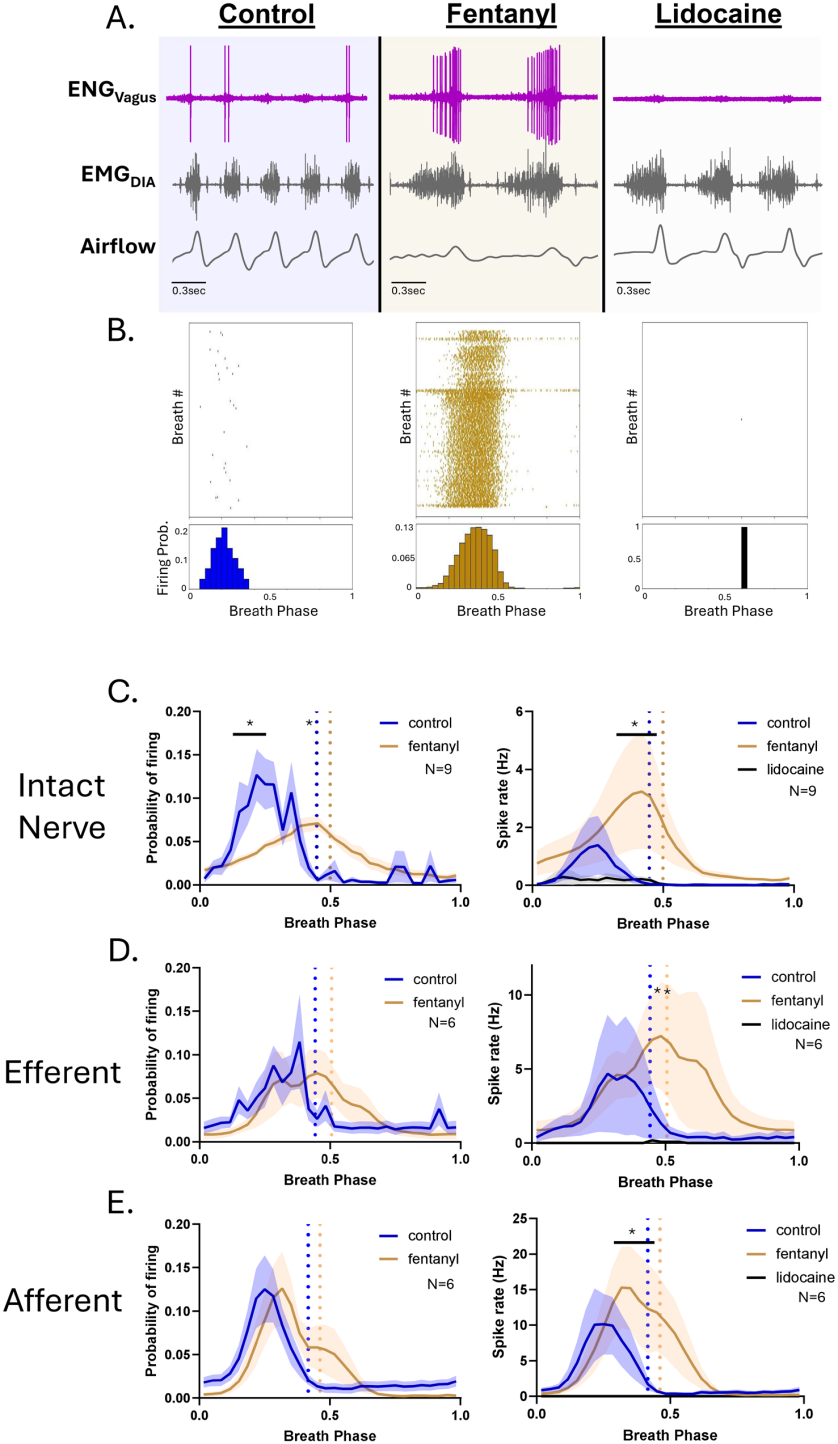
Vagus nerve activity is enhanced following fentanyl administration. (A) Representative traces from an intact vagus nerve showing ENG activity (ENG_Vagus_), diaphragm EMG (EMG_DIA_), and respiratory airflow during the control period and following fentanyl and lidocaine application. Fentanyl markedly increases spontaneous vagal spiking. (B) Raster plots showing spike activity across sequential breaths, with each row representing one breath. Below each raster is a histogram depicting the average probability of spike firing across the normalized respiratory cycle. (C, left) In the intact vagus, fentanyl broadens the temporal distribution of spikes, with activity extending beyond inspiration and into expiration (*N* = 9). (D, left) A similar expansion of firing is observed in the efferent branch following unilateral vagotomy (*N* = 6). (E, left) In contrast, afferent branch activity is less affected by fentanyl and remains more phase‐constrained (*N* = 6). (C–E, right) Firing rate histograms show that fentanyl increases spike rates across most phases of the respiratory cycle in the intact nerve (C), efferent branch (D), and afferent branch (E). Dotted vertical lines in panels C–E mark the average transition point between inspiration and expiration. * represents *p* < 0.05 for fentanyl period compared to control, two‐way repeated measures ANOVA with Holm–Šidák multiple comparisons, with factors for respiratory phase (binned across the normalized breath cycle) and drug condition (baseline, fentanyl, and lidocaine).

To examine the in vivo effects of an acute fentanyl overdose on vagus nerve activity, simultaneous electromyographic (EMG) and neural recordings were performed in urethane‐anesthetized mice administered an intraperitoneal injection of fentanyl (500 μg/kg). Vagus nerve activity was monitored for changes in both large‐amplitude, single‐unit action potentials (Figure 3) and tonic ENG activity (Figure 2A), represented by low‐amplitude, multiunit activity (MUA) that reflects the summated discharge of smaller, unsorted spikes occurring throughout the respiratory cycle. Recordings were obtained from intact nerves to assess overall physiological responses, as well as from isolated afferent and efferent segments following unilateral vagotomy to distinguish direction‐specific contributions.

In the intact nerve, the majority of animals (6/10) displayed a pronounced increase in tonic MUA following fentanyl administration. However, two animals showed a decrease in tonic activity, and another two exhibited no appreciable change (Figure 2B). In the cases showing increased activity, fentanyl initially caused a transient suppression of MUA, often resulting in near‐complete silencing for several seconds to a minute. This was followed by a robust increase in tonic discharge approximately 2–3 min after injection. The elevated MUA persisted for 10–15 min before gradually returning toward baseline. In half of these cases (3/6), tonic MUA had returned to near pre‐injection levels within 30–35 min. Across all animals that showed an increase, the average rise in tonic MUA was 43%, with some animals demonstrating more than a 20‐fold increase in integrated ENG activity (Figure 2B). In contrast, animals that exhibited a decrease in activity showed an average reduction of 22% in tonic MUA over the 30‐min post‐injection period.

To determine whether the fentanyl‐induced changes in tonic vagal activity were driven by central (efferent) or peripheral (afferent) mechanisms, we next performed unilateral vagotomy and recorded separately from the proximal (efferent) and distal (afferent) stumps of the transected nerve. In nearly all cases, afferent fibers showed a statistically significant (*p* = 0.01, *N* = 4), but minimal decrease in tonic ENG activity, with the exception of one animal that exhibited a large decline before stabilizing (Figure 2D). In contrast, efferent recordings more closely resembled those from intact nerves. In 50% of efferent recordings (4/8), tonic MUA increased within 2–3 min of fentanyl injection, followed by a gradual return to baseline across the 30‐min recording session (Figure 2C). On average, these efferent responses showed a 15% increase in tonic activity relative to baseline (*p* = 0.04). The remaining efferent cases (4/8) showed little or no change (*p* = 0.45), potentially reflecting variability in the specific subset of efferent fibers sampled with the hook electrode. Together, these results suggest that the majority of the fentanyl‐induced increase in tonic vagal activity is likely mediated through enhanced efferent discharge rather than direct changes in afferent signaling.

### 3.3 Fentanyl Enhances Excitability in Large‐Amplitude Single Unit Vagal Nerve Activity

To analyze large‐amplitude single‐unit activity from the vagus nerve, we employed a hook electrode system coupled with offline spike sorting techniques. Similar to the tonic MUA, recordings were obtained from urethane‐anesthetized mice using three configurations: whole‐nerve recordings (intact vagus), afferent nerve‐stump recordings (proximal to vagal transection), and efferent nerve‐stump recordings (distal to vagal transection). ENG signals were recorded during a baseline period and continuously for 30 min following intraperitoneal administration of fentanyl (500 μg/kg). At the conclusion of the fentanyl recording period, lidocaine was applied locally to the exposed vagus nerve to suppress nerve activity and isolate and eliminate electromyographic (EMG) and cardiac artifacts, which can often contaminate vagal recordings. Spike detection was performed using a threshold‐based approach, with the detection threshold set above any residual signal observed following lidocaine application to ensure that only true nerve activity was analyzed. Detected spikes were then projected into principal component (PC) space and clustered using template‐based sorting to isolate individual units. Unit quality was verified by visual inspection of waveform consistency and confirmation of a distinct refractory period in the interspike interval histogram, indicative of well‐isolated single neurons. Following spike sorting, large‐amplitude single‐unit vagal fiber activity was evaluated during acute fentanyl overdose (500 μg/kg, intraperitoneal). Given the strong respiratory modulation of vagal firing, spike activity was analyzed in relation to the respiratory phase, with phase 0 representing the onset of inspiration and phase 1 marking the beginning of the subsequent (*N* + 1) inspiratory effort.

Spike activity was characterized by both the overall firing rate and its phase distribution across the respiratory cycle, quantified as the probability of spike occurrence within a normalized breath phase. Prior to fentanyl administration, spontaneous spike activity was sparse and tightly constrained to inspiration, with nearly all spikes occurring within the first 25% of the respiratory cycle (Figure 3). Following fentanyl administration, however, single unit firing increased markedly, accompanied by a pronounced shift in phase distribution. Instead of being restricted to early inspiration, spike timing became broadly distributed, forming a near‐Gaussian pattern that extended throughout the entire respiratory cycle (Figure 3C–E). This expansion into expiration suggests that fentanyl disrupts the timing constraints normally imposed by central pattern‐generating networks, thereby diminishing the phase specificity of vagal discharge.

To determine whether these changes were driven by efferent or afferent contributions, we analyzed spike activity in the isolated efferent and afferent branches of the vagus nerve following unilateral transection (Figure 3D,E). Efferent activity closely mirrored the responses observed in intact nerves, with fentanyl inducing a significant increase in single‐unit firing and a broader phase distribution that extended well into expiration, a region previously devoid of large‐amplitude vagal spikes. In contrast, afferent fibers exhibited a more modest increase in spike rate following fentanyl, with only a minor shift in the timing of activity. Most afferent firing remained constrained to the inspiratory phase, although a slight rightward shift in the phase histogram was observed, consistent with the normal prolongation of inspiration induced by fentanyl (blue and gold dotted lines in Figure 3C–E).

Taken together, these findings indicate that fentanyl significantly enhances vagal single‐unit activity and disrupts the temporal fidelity of vagal discharge. This effect is largely mediated by increased efferent firing and reflects a breakdown in the respiratory phase‐locking normally maintained by central pattern‐generating circuits.

### 3.4 Fentanyl Induced Airway Obstructions Are Prevented by Vagotomy or Atropine

Previous studies have shown that fentanyl administration leads to a pronounced, transient airway obstruction, characterized by a delay between the onset of diaphragm EMG activity and the onset of inspiratory airflow [44]. To determine whether the heightened vagal activity observed following fentanyl contributes to this transient obstruction, we performed bilateral vagotomy prior to intraperitoneal injection of fentanyl (500 μg/kg). Additionally, in a separate group of mice, we co‐administered atropine (0.1 mg/kg I.P.) with fentanyl (500 μg/kg).

In sham‐operated control animals, fentanyl significantly increased the delay between the onset of diaphragm EMG activity and the onset of airflow by 865% ± 199% (*p* < 0.01, *N* = 6), replicating the characteristic transient obstruction. In contrast, vagotomized animals exhibited no delay following fentanyl administration (*p* = 0.87, *N* = 6), such that the timing between diaphragm activity and airflow onset remained unchanged. In animals co‐administered atropine and fentanyl, there was a miniscule, but statistically significant increase in the delay between the onset of diaphragm EMG activity and the onset of airflow (*p* = 0.032, *N* = 6). However, this delay was significantly less than the sham animals following fentanyl (*p* < 0.001, *N* = 6) and significantly less compared to animals given fentanyl following vagotomy (*p* = 0.01, *N* = 6). These results suggest that the fentanyl‐induced transient obstruction is mediated, at least in part, by vagal influence on the airways.

**FIGURE 4:**
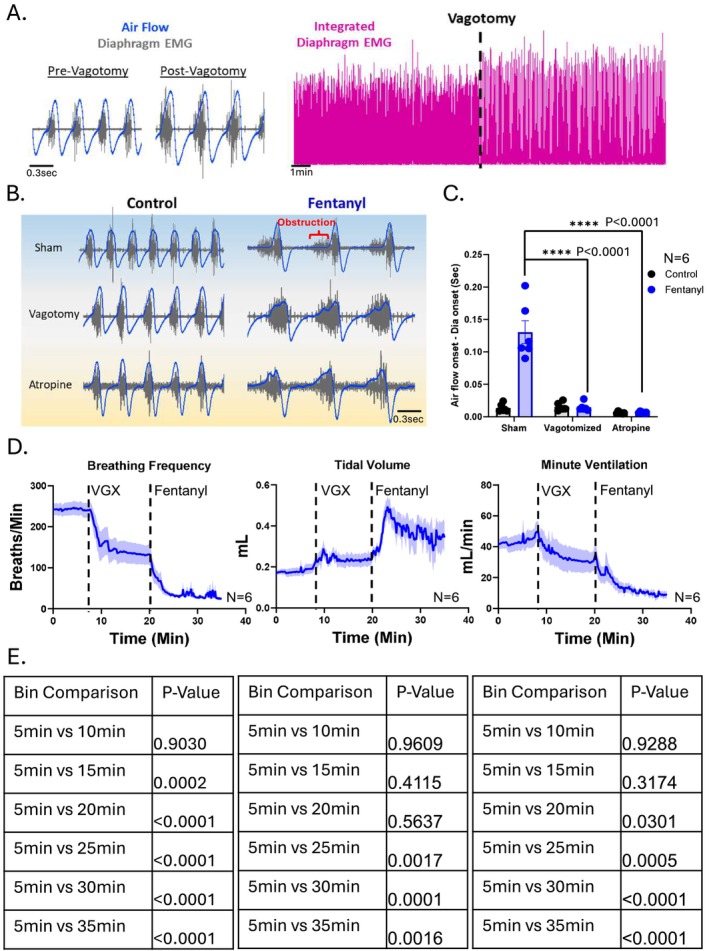
Bilateral vagotomy or atropine prevents fentanyl‐induced airflow obstruction. (A, left) Representative traces showing diaphragm EMG (gray) and respiratory airflow (blue) before and after bilateral vagotomy. (A, right) Compressed diaphragm EMG traces illustrate the characteristic physiological effect of vagotomy, an increase in inspiratory burst amplitude and a reduction in respiratory rate. (B) Representative traces from sham‐operated, vagotomized, and atropine administered mice showing diaphragm EMG and airflow before and after intraperitoneal fentanyl administration (500 μg/kg). In sham animals, fentanyl induced periodic airflow obstructions, evident as a delay between the onset of diaphragm activation and the initiation of inspiratory airflow. This delay was absent in vagotomized animals and animals administered atropine, highlighting the critical role of vagal input in contributing ot fentanyl‐induced airway obstruction. (C) Quantification of the latency between diaphragm EMG onset and inspiratory airflow initiation demonstrates that fentanyl significantly increased this delay in sham‐operated animals (*p* < 0.01, *N* = 6), whereas no such increase occurred in vagotomized mice (*p* = 0.87, *N* = 6). Animals co‐administered atropine with fentanyl displayed a miniscule, but significant increase in the diaphragm‐airflow delay (*p* = 0.032, *N* = 6). However, the delay following this increase was significantly lower than animals with sham or vagotomy. (D) Time course of respiratory parameters over the duration of the study. Vagotomy alone induced a classic reduction in breathing rate accompanied by a compensatory increase in tidal volume. Following fentanyl administration, both sham and vagotomized animals exhibited further reductions in breathing rate and increases in tidal volume. However, the net effect was a sustained reduction in overall minute ventilation. (E) 5‐min binned data for statistical analysis with a one‐way RM ANOVA. Bin times represent the end of the 5 min bin (ex. “5 min” = 0‐5 min). Baseline variables, prior to fentanyl administration are shown as “5 min” and “10 min”. * represents *p* < 0.05.

As expected, following vagotomy, animals also demonstrated a decrease in respiratory rate accompanied by a compensatory increase in tidal volume, consistent with the loss of vagal afferent input to central respiratory networks [51, 52]. Data in Figure 4D are plotted in 10‐s bins to illustrate the temporal pattern of respiratory suppression. For statistical analysis, ventilatory parameters were also averaged into 5‐min bins (Figure 4E), as shown in the accompanying table, to facilitate comparison across timepoints.

### 3.5 Relationship Between Vagal Activation and Partial Airway Obstruction

To determine whether there was a relationship between the degree of airflow obstruction and the magnitude of vagus nerve activation, we assessed the correlation between neural activity and the delay between diaphragm EMG onset and the onset of inspiratory airflow. Specifically, we calculated Spearman's rho to evaluate the association between single‐unit firing frequency and airflow delay across all large‐amplitude single units, as well as for tonic MUA.

**FIGURE 5:**
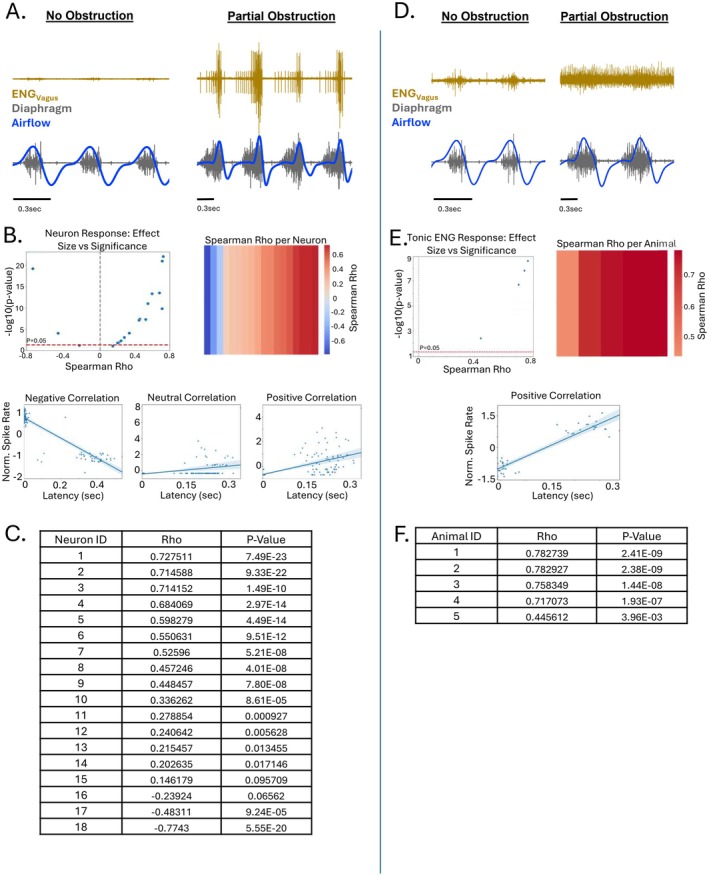
Vagus nerve activation correlates with the severity of airflow obstruction following fentanyl administration. (A) Representative traces showing vagal ENG activity (ENGVagus) alongside diaphragm EMG (EMG_DIA_) and respiratory airflow, highlighting large‐amplitude single‐unit spikes. Fentanyl administration results in increased spike activity and altered timing relative to inspiratory airflow. (B, top left) Volcano plot illustrating the Spearman's rho correlation coefficients between spike frequency of individual large‐amplitude vagal neurons and the degree of fentanyl‐induced airflow obstruction. Dots represent individual units, with significance indicated by *p*‐value thresholds. (B, top right) Heatmap showing the Spearman's rho values for the correlation between spike rate and airflow obstruction across all recorded large‐amplitude single units. (B, bottom) Example spike frequency profiles from three representative neurons that exhibited either a positive correlation (increased firing during obstruction), no change, or a negative correlation (decreased firing during obstruction), respectively. (C) Table displaying the Spearman's rho values and corresponding *p*‐values for each recorded neuron following fentanyl administration. (D) Representative traces of tonic multiunit activity (MUA) recorded from the vagus nerve, with diaphragm EMG and airflow shown before and after fentanyl administration. Tonic MUA increases in parallel with the emergence of airflow obstruction. (E, left) Volcano plot showing the relationship between tonic MUA amplitude and severity of airflow obstruction, derived from breath‐to‐breath correlations. (E, right) Heatmap showing the Spearman's rho correlation values between MUA activity and airflow obstruction across all animals. (F) Summary table showing the Spearman's rho values and *p*‐values for the correlation between tonic MUA and airflow obstruction in each animal. Together, these analyses reveal a strong and consistent relationship between increased vagal activity, particularly tonic MUA, and the degree of fentanyl‐induced airflow obstruction. In contrast, single‐unit responses were more heterogeneous, with some neurons increasing, decreasing, or showing no change in firing during obstruction.

Among large‐amplitude single units, we observed a broad distribution of correlation values. Some units exhibited strong positive correlations with airflow obstruction, with Spearman's rho values as high as +0.73, indicating that firing increased in parallel with greater obstruction. Conversely, other units showed significant negative correlations, with rho values as low as −0.77, suggesting that firing decreased as obstruction worsened (Figure 5B,C). These divergent relationships imply functional heterogeneity among single vagal fibers, with distinct subpopulations potentially contributing differentially to airway regulation during fentanyl‐induced disruption. A volcano plot of this analysis (Figure 5B) illustrates that units with the strongest correlations, both positive and negative, also had the most statistically significant associations, reinforcing the robustness of these relationships.

In contrast, analysis of tonic MUA showed a much more consistent pattern (Figure 5D–F). Across all animals, there was a positive correlation between MUA amplitude and the duration of airflow delay. Breaths with the longest latency between diaphragm activation and airflow onset consistently exhibited the greatest increases in tonic MUA. Moreover, animals displaying the largest overall increases in MUA were also those with the most severe obstructions. This uniform relationship suggests that while both large‐amplitude single units and tonic MUA may contribute to airflow obstruction, the tonic component, likely reflecting the summed activity of small, unsorted fibers, plays a more robust role in mediating the severity of the obstruction.

### 3.6 Intracisternal Naloxone Reverses Fentanyl‐Induced Airflow Obstruction

Our findings demonstrating increased vagal activity following fentanyl administration, along with the prevention of airflow obstruction by vagotomy and atropine, suggest that fentanyl‐induced recruitment of vagal firing plays a key role in mediating airway obstruction. Furthermore, our data showing that efferent vagal activity most closely mirrors the response observed in intact nerves indicates that this effect likely originates from enhanced central parasympathetic outflow.

**FIGURE 6:**
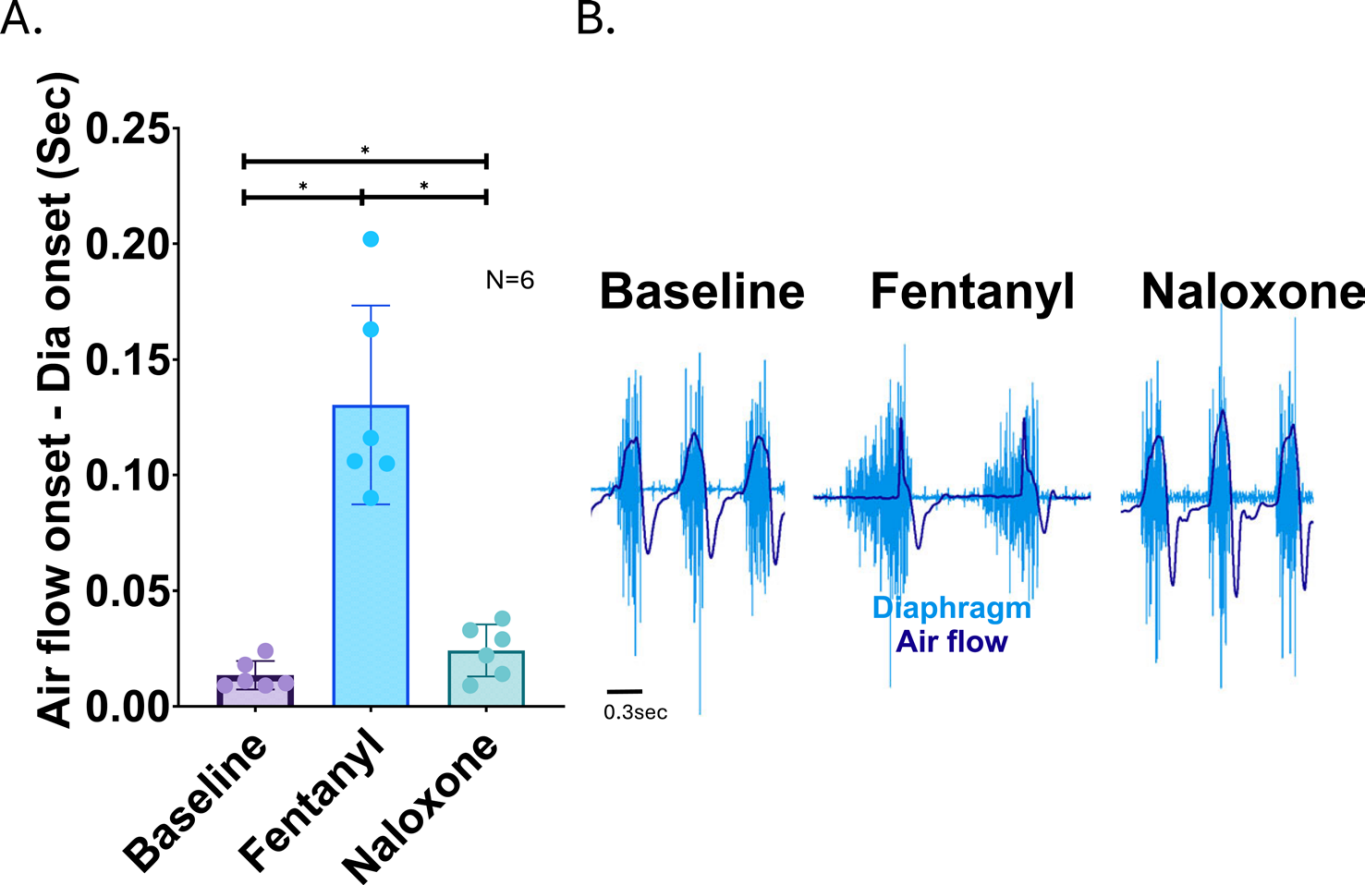
Intracisternal naloxone rapidly reverses fentanyl‐induced airflow obstruction. (A) Quantification of the latency between diaphragm EMG activation and the onset of inspiratory airflow during baseline, following intraperitoneal fentanyl administration (500 μg/kg), and after intracisternal injection of naloxone (10 mg/kg). Fentanyl significantly increases the diaphragm‐to‐airflow latency, reflecting a transient obstruction of inspiratory airflow (*N* = 6). This effect is rapidly and reliably reversed by central administration of naloxone, indicating a central, opioid receptor‐mediated mechanism underlying the obstruction. (B) Representative traces showing diaphragm EMG (blue) and respiratory airflow (purple) during control (baseline), following fentanyl, and after intracisternal naloxone. During fentanyl, there is a clear delay between the onset of diaphragm activity and the start of airflow, consistent with obstruction. This delay resolves immediately following naloxone injection, restoring normal coupling between inspiratory muscle activation and airflow. * represents *p* < 0.05.

To directly test whether blocking central opioid receptors could reverse fentanyl‐induced airflow obstruction, we administered intracisternal naloxone following the onset of obstruction caused by intraperitoneal injection of fentanyl (500 μg/kg). In all animals tested, intracisternal naloxone rapidly and reliably reversed the airway obstruction, restoring normal timing between diaphragm EMG activity and inspiratory airflow (Figure 6).

These results support the hypothesis that fentanyl enhances central parasympathetic efferent output via opioid receptor activation within the brainstem, leading to increased vagal tone and subsequent airway constriction. Central opioid receptor antagonism via intracisternal naloxone is sufficient to counteract this effect, highlighting a key brainstem‐mediated mechanism underlying fentanyl‐induced respiratory dysfunction.

## 4 Discussion

The current study provides new mechanistic insight into how fentanyl impairs respiration, extending the understanding of opioid‐induced respiratory depression (OIRD) beyond its well‐characterized effects on rhythm generation. Our findings support a model in which fentanyl enhances central parasympathetic outflow through the vagus nerve, resulting in transient but physiologically significant airway obstruction. This obstruction is not due to respiratory rhythm suppression alone, but reflects a breakdown in the coordination between inspiratory muscle activity and effective airflow, evidenced by a delay between diaphragm EMG activation and the onset of inspiratory airflow.

Previous work, including our own [44], has shown that fentanyl induces transient airway obstruction during overdose, characterized by a measurable delay between diaphragm contraction and airflow initiation. Prior studies have further suggested that these obstructions may involve active constriction of airway smooth muscle, given that they can be reversed by bronchodilators such as salbutamol or epinephrine [44, 53]. Building on this, our findings demonstrate that fentanyl‐induced airway obstruction may, in part, be dependent on vagus nerve activity. Fentanyl significantly increases vagal output, as evidenced by elevated tonic multiunit activity (MUA) and enhanced large‐amplitude single‐unit firing, particularly within efferent fibers. These changes closely correlate with the severity of airflow obstruction. Notably, bilateral vagotomy or atropine administration completely abolished the fentanyl‐induced delay between diaphragm activation and airflow onset, indicating that vagal signaling, not peripheral muscular rigidity alone, is essential for generating the obstructive phenotype.

The vagus nerve exerts both afferent and efferent control over airway tone [38, 54, 55]. Vagal afferents arising from the nodose and jugular ganglia detect stretch, inflammation, and chemical irritation and project centrally to the nucleus tractus solitarius (NTS) [22, 54]. Vagal efferents, originating primarily from the nucleus ambiguus and to a lesser extent from the dorsal motor nucleus of the vagus (DMV), project to parasympathetic ganglia embedded within airway walls [19, 33, 56]. These postganglionic fibers release acetylcholine, primarily acting on M3 muscarinic receptors on airway smooth muscle (ASM) to promote bronchoconstriction [34, 57]. While M2 receptors are more abundant in ASM, they predominantly function at presynaptic sites to inhibit acetylcholine release, thereby modulating but not directly controlling airway tone.

Critically, μ‐opioid receptors (MORs) are densely expressed throughout the brainstem in regions controlling vagal output, including the nucleus ambiguus, DMV, and NTS, as well as on vagal afferent and efferent fibers. Prior studies have shown that MOR activation in these areas can result in complex excitatory effects depending on the circuit context [58, 59]. For example, fentanyl may disinhibit parasympathetic output by suppressing local GABAergic or glycinergic interneurons that normally inhibit vagal preganglionic neurons [60].

**FIGURE 7:**
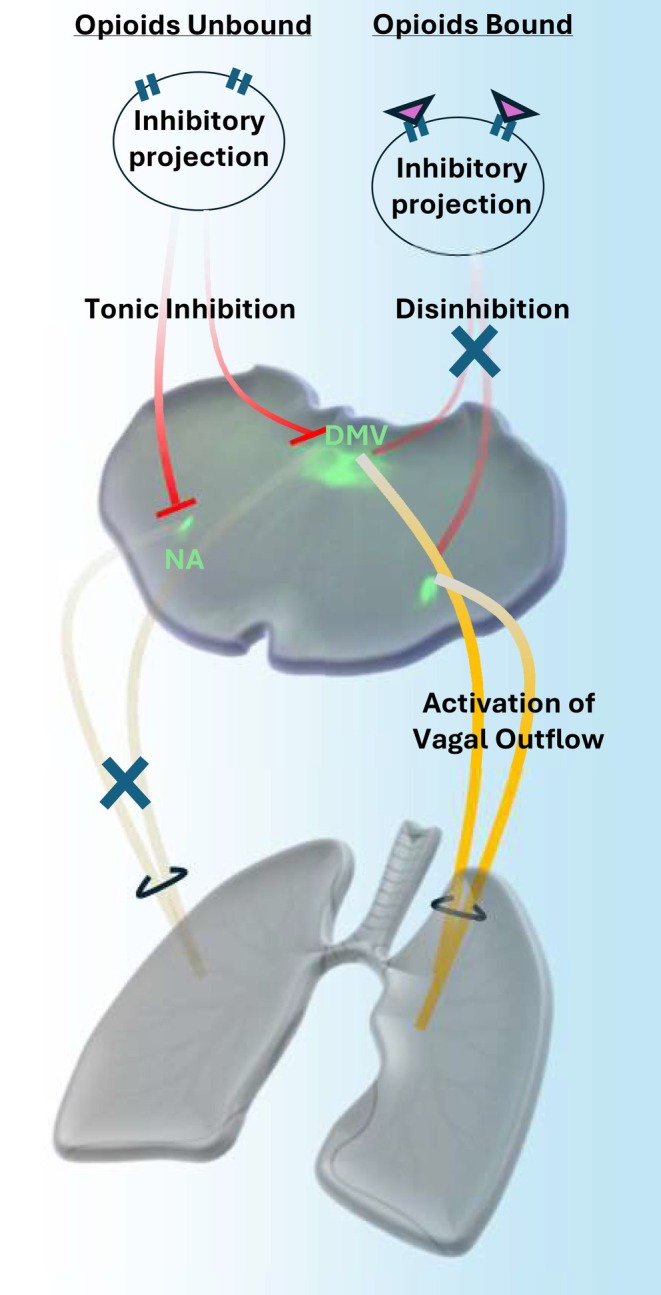
Hypothetical reduced circuit diagram of fentanyl‐mediated vagus nerve overactivation. Fentanyl administration results in an increase in vagus nerve activity that may contribute to airway disruption. A proposed mechanism is disinhibition of central parasympathetic motor nuclei, including the dorsal motor nucleus of the vagus (DMV) and nucleus ambiguus (NA). Under control conditions, opioid‐sensitive tonic inhibition may dampen parasympathetic motor drive (left). We hypothesize that opioids inhibit this tonic inhibition, thereby overactivating vagal motor output (right). The coronal brainstem slice shown was obtained from a ChAT‐Cre; Ai32 mouse (Chat^tm2(cre)Lowl; Gt(ROSA)26Sor^tm32(CAG‐COP4*H134R/EYFP)Hze) (Jax Labs), in which parasympathetic motoneurons are labeled with GFP.

Additionally, recent work on spinal cord phrenic motoneurons suggests that fentanyl may exert direct excitatory effects via noncanonical mechanisms, such as blockade of voltage‐gated potassium channels (Kv), which can increase motoneuron excitability [61]. Our findings support a centrally mediated, opioid receptor‐dependent mechanism for fentanyl‐induced airway obstruction. The rapid and reliable reversal of obstruction by intracisternal naloxone, an antagonist that selectively blocks μ‐opioid receptors, strongly implicates brainstem MOR activation as the primary driver of vagal overactivation. Additionally, the observed increase in tonic vagal activity and the loss of respiratory phase‐locking in large‐amplitude single‐unit firing, particularly in efferent fibers, suggest that fentanyl acts within central parasympathetic circuits to enhance vagal output. Importantly, these effects are unlikely to result from non‐receptor‐mediated mechanisms such as direct Kv channel inhibition, as such actions would not be reversed by naloxone. Together, these results indicate that fentanyl contributes to airflow disruption by engaging central opioid receptors that disinhibit or amplify parasympathetic drive to the airways, rather than through direct peripheral excitatory effects (Figure 7).

Beyond increasing overall vagal output, fentanyl also disrupts the precise temporal coordination of vagal firing relative to the respiratory cycle, an essential feature of autonomic‐respiratory integration [62]. Notably, fentanyl administration dramatically altered the timing and distribution of single‐unit vagal activity. Under control conditions, large‐amplitude vagal spikes were phase‐locked to the inspiratory phase, with minimal firing during expiration. This timing likely reflects the respiratory‐coupled role of parasympathetic efferents in modulating airway tone in synchrony with ventilation. After fentanyl, however, single‐unit firing became desynchronized, with activity extending across the entire respiratory cycle, including expiration, a phase typically devoid of efferent activity. This temporal disorganization suggests fentanyl disrupts the phase‐specific coordination of brainstem circuits responsible for integrating respiratory rhythm with autonomic output.

Such disruption may arise from fentanyl's actions within the preBötzinger complex, NTS, or reticular formation, which integrate afferent input and drive both somatic and autonomic respiratory outputs. Desynchronized parasympathetic outflow could lead to inappropriately timed bronchoconstriction during expiration or at the transition between respiratory phases, interfering with normal airflow mechanics [63]. These persistent but dysregulated descending signals may contribute to continued vagal outflow in the absence of coherent respiratory timing.

The functional consequence of this disorganization is significant. Parasympathetic activity during expiration could result in sustained or premature bronchoconstriction, increasing airway resistance when the airway should be relaxed. This mismatch, particularly when compounded by the thoracic and laryngeal rigidity associated with Wooden Chest Syndrome (WCS), may make patients unventilatable during overdose, even when the respiratory rhythm is restored. Importantly, these neural contributions to airway dysfunction are unlikely to be detectable using standard clinical metrics, highlighting the critical role of neural recording in revealing the full extent of fentanyl's impact on respiration.

Importantly, the relationship between vagal activation and obstruction was not uniform across all recorded units. Our volcano plot analysis revealed that while some large‐amplitude fibers exhibited strong positive correlations between firing rate and obstruction severity, others showed negative or no relationship. This heterogeneity suggests the presence of distinct functional subpopulations within the vagus, some potentially inhibitory or modulatory in airway control [64]. By contrast, the correlation between tonic MUA and airflow delay was remarkably consistent across animals. Given that MUA reflects low‐amplitude, small fibers, and that efferent activity was preserved in these signals post‐vagotomy, these findings indicate that small‐diameter parasympathetic efferents, including potentially unmyelinated fibers, play a dominant role in driving fentanyl‐induced obstruction.

One limitation of the present study is that afferent versus efferent contributions were distinguished by nerve transection with subsequent recordings from the proximal and distal stumps. While this approach provides strong directionality by physically isolating traffic, it may also introduce confounds such as autonomous activity in residual fibers or secondary effects related to nerve injury and inflammation. Future studies could utilize reversible strategies, such as multi‐point cuff electrodes or anodal/depolarization block techniques, which have been used successfully to selectively modulate afferent and efferent traffic [65, 66, 67, 68]. These refinements would reduce the potential impact of injury‐related artifacts and further sharpen the specificity of the findings.

Together, these results have translational implications. Current resuscitation strategies in fentanyl overdose emphasize opioid antagonism and mechanical ventilation [69, 70, 71, 72], yet many patients remain unresponsive to bag‐mask ventilation due to airway obstruction and chest wall rigidity. While rigidity clearly contributes, our data indicate a central neurogenic component involving vagally mediated airway disruption. Therapeutic interventions that modulate vagal output, such as muscarinic antagonists (e.g., atropine, ipratropium), β2‐agonists (e.g., albuterol), or centrally acting opioid antagonists, could provide critical adjunctive benefit during overdose reversal.

In summary, this study provides direct in vivo evidence that fentanyl enhances central parasympathetic efferent output, disrupts respiratory phase‐specific firing in the vagus nerve, and induces transient, reversible airway disruption. These effects are mediated through central opioid mechanisms, can be prevented by vagotomy or atropine, and reversed by intracisternal naloxone. Understanding this neurogenic contribution to fentanyl‐induced airway dysfunction opens new avenues for targeted therapeutic strategies aimed at reducing opioid overdose mortality.

## Author Contributions

R.R.P., M.J.A., and N.J.B.: conceptualization. R.R.P., N.J.B.: methodology, investigation, formal analysis. R.R.P., M.J.A., and N.J.B.: writing – review and editing. N.J.B.: supervision and funding acquisition.

## Conflicts of Interest

The authors declare no conflicts of interest.

## Data Availability

The data that support the findings of this study are available from the corresponding author upon reasonable request.

## References

[1] L. Scholl , P. Seth , M. Kariisa , N. Wilson , and G. Baldwin , “Drug and Opioid‐Involved Overdose Deaths—United States, 2013–2017,” Morbidity and Mortality Weekly Report 67, no. 5152 (2018): 1419–1427. 30605448 10.15585/mmwr.mm675152e1 PMC6334822

[2] B. A. Baldo and M. A. Rose , “Mechanisms of Opioid‐Induced Respiratory Depression,” Archives of Toxicology 96, no. 8 (2022): 2247–2260. 35471232 10.1007/s00204-022-03300-7

[3] B. A. Baldo , “Toxicities of Opioid Analgesics: Respiratory Depression, Histamine Release, Hemodynamic Changes, Hypersensitivity, Serotonin Toxicity,” Archives of Toxicology 95, no. 8 (2021): 2627–2642. 33974096 10.1007/s00204-021-03068-2

[4] R. Hill , R. Santhakumar , W. Dewey , E. Kelly , and G. Henderson , “Fentanyl Depression of Respiration: Comparison With Heroin and Morphine,” British Journal of Pharmacology 177, no. 2 (2020): 254–266. 31499594 10.1111/bph.14860 PMC6989952

[5] J. M. Ramirez , N. J. Burgraff , A. D. Wei , et al., “Neuronal Mechanisms Underlying Opioid‐Induced Respiratory Depression: Our Current Understanding,” Journal of Neurophysiology 125, no. 5 (2021): 1899–1919. 33826874 10.1152/jn.00017.2021 PMC8424565

[6] S. Liu , D. I. Kim , T. G. Oh , et al., “Neural Basis of Opioid‐Induced Respiratory Depression and Its Rescue,” Proceedings of the National Academy of Sciences of the United States of America 118, no. 23 (2021): 118. 10.1073/pnas.2022134118 PMC8201770 34074761

[7] M. Niesters , F. Overdyk , T. Smith , L. Aarts , and A. Dahan , “Opioid‐Induced Respiratory Depression in Paediatrics: A Review of Case Reports,” British Journal of Anaesthesia 110, no. 2 (2013): 175–182. 23248093 10.1093/bja/aes447

[8] B. Palkovic , V. Marchenko , E. J. Zuperku , E. A. Stuth , and A. G. Stucke , “Multi‐Level Regulation of Opioid‐Induced Respiratory Depression,” Physiology (Bethesda) 35, no. 6 (2020): 391–404. 33052772 10.1152/physiol.00015.2020 PMC7864237

[9] R. van der Schier , M. Roozekrans , M. Van Velzen , A. Dahan , and M. Niesters , “Opioid‐Induced Respiratory Depression: Reversal by Non‐Opioid Drugs,” F1000Prime Rep 6 (2014): 79. 25343036 10.12703/P6-79 PMC4173639

[10] A. Dahan , L. Aarts , and T. W. Smith , “Incidence, Reversal, and Prevention of Opioid‐Induced Respiratory Depression,” Anesthesiology 112, no. 1 (2010): 226–238. 20010421 10.1097/ALN.0b013e3181c38c25

[11] U. Baruah , H. Gaur , D. Saigal , and D. Pandey , “Wooden Chest Syndrome: A Curious Case of Fentanyl Induced Rigidity in Adults,” Indian Journal of Anaesthesia 66, no. 12 (2022): 881–882. 36654889 10.4103/ija.ija_171_22 PMC9842087

[12] F. L. Grell , R. A. Koons , and J. S. Denson , “Fentanyl in Anesthesia: A Report of 500 Cases,” Anesthesia and Analgesia 49, no. 4 (1970): 523–532. 5534663

[13] P. Haouzi and N. Tubbs , “Effects of Fentanyl Overdose‐Induced Muscle Rigidity and Dexmedetomidine on Respiratory Mechanics and Pulmonary Gas Exchange in Sedated Rats,” Journal of Applied Physiology 132, no. 6 (1985): 1407–1422. 10.1152/japplphysiol.00819.2021 PMC9190730 35421320

[14] N. R. Rosal , F. L. Thelmo, Jr. , S. Tzarnas , L. DiCalvo , S. Tariq , and C. Grossman , “Wooden Chest Syndrome: A Case Report of Fentanyl‐Induced Chest Wall Rigidity,” Journal of Investigative Medicine High Impact Case Reports 9 (2021): 34036. 10.1177/23247096211034036 PMC8312149 34301155

[15] F. L. Scamman , “Fentanyl‐O_2_‐N_2_O Rigidity and Pulmonary Compliance,” Anesthesia and Analgesia 62, no. 3 (1983): 332–334. 6829933

[16] J. B. Streisand , P. L. Bailey , L. LeMaire , et al., “Fentanyl‐Induced Rigidity and Unconsciousness in Human Volunteers. Incidence, Duration, and Plasma Concentrations,” Anesthesiology 78, no. 4 (1993): 629–634. 8466061 10.1097/00000542-199304000-00003

[17] B. J. Canning and A. Fischer , “Neural Regulation of Airway Smooth Muscle Tone,” Respiration Physiology 125, no. 1–2 (2001): 113–127. 11240156 10.1016/s0034-5687(00)00208-5

[18] A. E. McGovern and S. B. Mazzone , “Characterization of the Vagal Motor Neurons Projecting to the Guinea Pig Airways and Esophagus,” Frontiers in Neurology 1 (2010): 153. 21188271 10.3389/fneur.2010.00153 PMC3007679

[19] D. Jordan , “Central Nervous Pathways and Control of the Airways,” Respiration Physiology 125, no. 1–2 (2001): 67–81. 11240153 10.1016/s0034-5687(00)00205-x

[20] S. B. Mazzone and B. J. Canning , “Central Nervous System Control of the Airways: Pharmacological Implications,” Current Opinion in Pharmacology 2, no. 3 (2002): 220–228. 12020461 10.1016/s1471-4892(02)00151-0

[21] J. J. Fontán , C. T. Diec , and C. R. Velloff , “Bilateral Distribution of Vagal Motor and Sensory Nerve Fibers in the Rat's Lungs and Airways,” American Journal of Physiology. Regulatory, Integrative and Comparative Physiology 279, no. 2 (2000): R713–R728. 10938263 10.1152/ajpregu.2000.279.2.R713

[22] S. L. Prescott and S. D. Liberles , “Internal Senses of the Vagus Nerve,” Neuron 110, no. 4 (2022): 579–599. 35051375 10.1016/j.neuron.2021.12.020 PMC8857038

[23] J. Kim and B. Undem , Primer on the Autonomic Nervous System, Fourth ed. (Academic Press, 2023).

[24] R. Behrens , A. E. McGovern , M. J. Farrell , A. A. K. Moe , and S. B. Mazzone , “Mini Review: Central Organization of Airway Afferent Nerve Circuits,” Neuroscience Letters 744 (2021): 135604. 33387662 10.1016/j.neulet.2020.135604

[25] S. B. Mazzone and B. J. Undem , “Vagal Afferent Innervation of the Airways in Health and Disease,” Physiological Reviews 96, no. 3 (2016): 975–1024. 27279650 10.1152/physrev.00039.2015 PMC4982036

[26] L. Kubin , “Central Pathways of Pulmonary and Lower Airway Vagal Afferents,” Journal of Applied Physiology 101, no. 2 (1985): 618–627. 10.1152/japplphysiol.00252.2006 PMC4503231 16645192

[27] J. Li and Y. Liu , “Vagal Sensory Circuits of the Lower Airway in Respiratory Physiology: Insights From Neuronal Diversity,” Current Opinion in Neurobiology 92 (2025): 103000. 40101474 10.1016/j.conb.2025.103000

[28] J. G. Widdicombe , “Neurophysiology of the Cough Reflex,” European Respiratory Journal 8, no. 7 (1995): 1193–1202. 7589405 10.1183/09031936.95.08071193

[29] B. J. Undem , M. J. Carr , and M. Kollarik , “Physiology and Plasticity of Putative Cough Fibres in the Guinea Pig,” Pulmonary Pharmacology & Therapeutics 15, no. 3 (2002): 193–198. 12099763 10.1006/pupt.2002.0362

[30] J. Widdicombe , “Control of Airway Caliber,” American Review of Respiratory Disease 131, no. 5 (1985): S33–S35. 4003906 10.1164/arrd.1985.131.S5.S33

[31] D. B. Hill , B. Button , M. Rubinstein , and R. C. Boucher , “Physiology and Pathophysiology of Human Airway Mucus,” Physiological Reviews 102, no. 4 (2022): 1757–1836. 35001665 10.1152/physrev.00004.2021 PMC9665957

[32] M. A. Haxhiu , A. S. P. Jansen , N. S. Cherniack , and A. D. Loewy , “CNS Innervation of Airway‐Related Parasympathetic Preganglionic Neurons: A Transneuronal Labeling Study Using Pseudorabies Virus,” Brain Research 618, no. 1 (1993): 115–134. 8402166 10.1016/0006-8993(93)90435-p

[33] S. Hadziefendic and M. A. Haxhiu , “CNS Innervation of Vagal Preganglionic Neurons Controlling Peripheral Airways: A Transneuronal Labeling Study Using Pseudorabies Virus,” Journal of the Autonomic Nervous System 76, no. 2–3 (1999): 135–145. 10412837 10.1016/s0165-1838(99)00020-x

[34] A. D. Fryer and D. B. Jacoby , “Muscarinic Receptors and Control of Airway Smooth Muscle,” American Journal of Respiratory and Critical Care Medicine 158 (1998): S154–S160. 9817739 10.1164/ajrccm.158.supplement_2.13tac120

[35] C. A. Hirshman , B. Lande , and T. L. Croxton , “Role of M2 Muscarinic Receptors in Airway Smooth Muscle Contraction,” Life Sciences 64, no. 6–7 (1999): 443–448. 10069508 10.1016/s0024-3205(98)00586-4

[36] P. J. Barnes , “Muscarinic Receptor Subtypes in Airways,” Life Sciences 52, no. 5–6 (1993): 521–527. 8441331 10.1016/0024-3205(93)90310-y

[37] M. S. Schappe , P. A. Brinn , N. R. Joshi , et al., “A Vagal Reflex Evoked by Airway Closure,” Nature 627, no. 8005 (2024): 830–838. 38448588 10.1038/s41586-024-07144-2 PMC10972749

[38] B. J. Canning , “Reflex Regulation of Airway Smooth Muscle Tone,” Journal of Applied Physiology 101, no. 3 (1985): 971–985. 10.1152/japplphysiol.00313.2006 16728519

[39] S. Nomura , Y. Q. Ding , T. Kaneko , J. L. Li , and N. Mizuno , “Localization of Mu‐Opioid Receptor‐Like Immunoreactivity in the Central Components of the Vagus Nerve: A Light and Electron Microscope Study in the Rat,” Neuroscience 73, no. 1 (1996): 277–286. 8783249 10.1016/0306-4522(96)00027-9

[40] J. Zhuang , X. Gao , S. Shi , and F. Xu , “Intravenous Bolus Injection of Fentanyl Triggers an Immediate Central and Upper Airway Obstructive Apnea via Activating Vagal Sensory Afferents,” Journal of Applied Physiology 137, no. 6 (1985): 1666–1677. 10.1152/japplphysiol.00614.2024 PMC11687853 39417800

[41] S. A. Aicher , A. Goldberg , S. Sharma , and V. M. Pickel , “Mu‐Opioid Receptors Are Present in Vagal Afferents and Their Dendritic Targets in the Medial Nucleus Tractus Solitarius,” Journal of Comparative Neurology 422, no. 2 (2000): 181–190. 10842226 10.1002/(sici)1096-9861(20000626)422:2<181::aid-cne3>3.0.co;2-g

[42] R. Cohendy , J. Y. Lefrant , M. Laracine , T. Rebiere , and J. J. Eledjam , “Effect of Fentanyl on Ventilatory Resistances During Barbiturate General Anaesthesia,” British Journal of Anaesthesia 69, no. 6 (1992): 595–598. 1361357 10.1093/bja/69.6.595

[43] M. Hajiha , M. A. DuBord , H. Liu , and R. L. Horner , “Opioid Receptor Mechanisms at the Hypoglossal Motor Pool and Effects on Tongue Muscle Activity In Vivo,” Journal of Physiology 587, no. 11 (2009): 2677–2692. 19403616 10.1113/jphysiol.2009.171678 PMC2714030

[44] N. J. Burgraff , N. A. Baertsch , and J. M. Ramirez , “A Comparative Examination of Morphine and Fentanyl: Unravelling the Differential Impacts on Breathing and Airway Stability,” Journal of Physiology 601, no. 20 (2023): 4625–4642. 37778015 10.1113/JP285163 PMC13059524

[45] E. N. Nicolai , M. L. Settell , B. E. Knudsen , et al., “Sources of Off‐Target Effects of Vagus Nerve Stimulation Using the Helical Clinical Lead in Domestic Pigs,” Journal of Neural Engineering 17, no. 4 (2020): 46017. 10.1088/1741-2552/ab9db8 PMC7717671 32554888

[46] H. A. Silverman , A. Stiegler , T. Tsaava , et al., “Standardization of Methods to Record Vagus Nerve Activity in Mice,” Bioelectronic Medicine 4 (2018): 3. 32232079 10.1186/s42234-018-0002-y PMC7098227

[47] A. Furdui , C. da Silveira Scarpellini , and G. Montandon , “Fentanyl‐Induced Respiratory Depression and Locomotor Hyperactivity Are Mediated by μ‐Opioid Receptors Expressed in Somatostatin‐Negative Neurons,” eNeuro 10, no. 6 (2023): ENEURO.0035–ENEU23.2023. 37364996 10.1523/ENEURO.0035-23.2023 PMC10312122

[48] A. Dahan , A. Yassen , H. Bijl , et al., “Comparison of the Respiratory Effects of Intravenous Buprenorphine and Fentanyl in Humans and Rats,” British Journal of Anaesthesia 94, no. 6 (2005): 825–834. 15833777 10.1093/bja/aei145

[49] E. A. Kiyatkin , “Respiratory Depression and Brain Hypoxia Induced by Opioid Drugs: Morphine, Oxycodone, Heroin, and Fentanyl,” Neuropharmacology 151 (2019): 219–226. 30735692 10.1016/j.neuropharm.2019.02.008 PMC6500744

[50] S. E. Saunders , D. M. Baekey , and E. S. Levitt , “Fentanyl Effects on Respiratory Neuron Activity in the Dorsolateral Pons,” Journal of Neurophysiology 128, no. 5 (2022): 1117–1132. 36197016 10.1152/jn.00113.2022 PMC9621704

[51] F. J. Golder , D. D. Fuller , P. W. Davenport , R. D. Johnson , P. J. Reier , and D. C. Bolser , “Respiratory Motor Recovery After Unilateral Spinal Cord Injury: Eliminating Crossed Phrenic Activity Decreases Tidal Volume and Increases Contralateral Respiratory Motor Output,” Journal of Neuroscience 23, no. 6 (2003): 2494–2501. 12657710 10.1523/JNEUROSCI.23-06-02494.2003 PMC6742041

[52] M. Kashani and A. L. Haigh , “The Effects of Vagotomy on Ventilation and Blood Gas Composition in Dog, Sheep and Rabbit,” Quarterly Journal of Experimental Physiology and Cognate Medical Sciences 60, no. 4 (1975): 285–298. 1041799 10.1113/expphysiol.1975.sp002322

[53] B. A. Baldo , “Opening the Door to Physiological Impacts Underlying Opioid‐Induced Respiratory Depression and Therapeutic Strategies: Focus on Fentanyl,” Journal of Physiology 601, no. 20 (2023): 4473–4474. 37738097 10.1113/JP285619

[54] R. B. Chang , D. E. Strochlic , E. K. Williams , B. D. Umans , and S. D. Liberles , “Vagal Sensory Neuron Subtypes That Differentially Control Breathing,” Cell 161, no. 3 (2015): 622–633. 25892222 10.1016/j.cell.2015.03.022 PMC4842319

[55] M. J. Carr and B. J. Undem , “Bronchopulmonary Afferent Nerves,” Respirology 8, no. 3 (2003): 291–301. 14528878 10.1046/j.1440-1843.2003.00473.x

[56] K. J. Audrit , L. Delventhal , Ö. Aydin , and C. Nassenstein , “The Nervous System of Airways and Its Remodeling in Inflammatory Lung Diseases,” Cell and Tissue Research 367, no. 3 (2017): 571–590. 28091773 10.1007/s00441-016-2559-7

[57] E. Roux , M. Molimard , and R. Marthan , “Muscarinic Stimulation of Airway Smooth Muscle Cells,” General Pharmacology 31, no. 3 (1998): 349–356. 9703200 10.1016/s0306-3623(98)00007-x

[58] P. M. Lalley , “Mu‐Opioid Receptor Agonist Effects on Medullary Respiratory Neurons in the Cat: Evidence for Involvement in Certain Types of Ventilatory Disturbances,” American Journal of Physiology. Regulatory, Integrative and Comparative Physiology 285, no. 6 (2003): R1287–R1304. 12881202 10.1152/ajpregu.00199.2003

[59] M. Laubie and H. Schmitt , “Action of the Morphinometic Agent, Fentanyl, on the Nucleas Tractus Solitarii and the Nucleus Ambiguus Cardiovascular Neurons,” European Journal of Pharmacology 67, no. 4 (1980): 403–412. 7449824 10.1016/0014-2999(80)90181-8

[60] K. J. Griffioen , P. Venkatesan , Z. G. Huang , et al., “Fentanyl Inhibits GABAergic Neurotransmission to Cardiac Vagal Neurons in the Nucleus Ambiguus,” Brain Research 1007, no. 1–2 (2004): 109–115. 15064141 10.1016/j.brainres.2004.02.010

[61] A. D. Wei , “Fentanyl Blockade of K,” bioRxiv (2025).

[62] F. Krohn , M. Novello , R. S. van der Giessen , C. I. De Zeeuw , J. J. Pel , and L. W. Bosman , “The Integrated Brain Network That Controls Respiration,” eLife 12 (2023): e83654. 36884287 10.7554/eLife.83654 PMC9995121

[63] E. R. Bleecker , “Cholinergic and Neurogenic Mechanisms in Obstructive Airways Disease,” American Journal of Medicine 81, no. 5A (1986): 93–102. 10.1016/0002-9343(86)90470-5 2878614

[64] K. Inoue , L. F. Samodelov , and J. O. Arndt , “Fentanyl Activates a Particular Population of Vagal Efferents Which Are Cardioinhibitory,” Naunyn‐Schmiedeberg's Archives of Pharmacology 312, no. 1 (1980): 57–61. 7393348 10.1007/BF00502575

[65] L. Joseph and R. J. Butera , “High‐Frequency Stimulation Selectively Blocks Different Types of Fibers in Frog Sciatic Nerve,” IEEE Transactions on Neural Systems and Rehabilitation Engineering 19, no. 5 (2011): 550–557. 21859632 10.1109/TNSRE.2011.2163082 PMC3308706

[66] Y. A. Patel , A. Willsie , I. P. Clements , R. Aguilar , S. Rajaraman , and R. J. Butera , “Microneedle Cuff Electrodes for Extrafascicular Peripheral Nerve Interfacing,” Annual International Conference of the IEEE Engineering in Medicine and Biology Society (EMBC) 2016 (2016): 1741–1744. 28268663 10.1109/EMBC.2016.7591053

[67] Y. A. Patel and R. J. Butera , “Challenges Associated With Nerve Conduction Block Using Kilohertz Electrical Stimulation,” Journal of Neural Engineering 15, no. 3 (2018): 031002. 29415877 10.1088/1741-2552/aaadc0

[68] A. Fitchett , S. Mastitskaya , and K. Aristovich , “Selective Neuromodulation of the Vagus Nerve,” Frontiers in Neuroscience 15 (2021): 685872. 34108861 10.3389/fnins.2021.685872 PMC8180849

[69] A. Robinson and D. P. Wermeling , “Intranasal Naloxone Administration for Treatment of Opioid Overdose,” American Journal of Health‐System Pharmacy 71, no. 24 (2014): 2129–2135. 25465584 10.2146/ajhp130798

[70] T. I. Saari , J. Strang , and O. Dale , “Clinical Pharmacokinetics and Pharmacodynamics of Naloxone,” Clinical Pharmacokinetics 63, no. 4 (2024): 397–422. 38485851 10.1007/s40262-024-01355-6 PMC11052794

[71] A. O. Oladunjoye , O. O. Oladunjoye , O. Olubiyi , M. R. Yee , and E. D. Espiridion , “Predictors and Outcomes of Invasive Mechanical Ventilation in Opioid Overdose Hospitalization in the United States,” Cureus 12, no. 8 (2020): e9788. 32953304 10.7759/cureus.9788 PMC7491682

[72] G. J. Pfister , R. M. Burkes , B. Guinn , et al., “Opioid Overdose Leading to Intensive Care Unit Admission: Epidemiology and Outcomes,” Journal of Critical Care 35 (2016): 29–32. 27481733 10.1016/j.jcrc.2016.04.022

